# Supplementary material for: Association between Dietary Habit and Clinical Parameters in Patients with Chronic Periodontitis Undergoing Supportive Periodontal Therapy
Source: Nutrients. 2022 Nov 24;14(23):4993. doi: 10.3390/nu14234993 (PMC9741307; doi:10.3390/nu14234993)

Table S1. Profiles of the clinical parameters in four stages

Objects		106						
Sex	Male	48						
	Female	58						
Stage of periodontal therapy		BL	RE	SPT1	SPT2	Differences		
	Period (days)	0	328 (13–2761)	876 (131–3966)	2935 (334–5602)	Between	Between	Between
Age		64 (37–84)	65 (39–84)	67 (43–86)	73 (44–88)	BL vs. SPT2	RE vs. SPT2	SPT1 vs. SPT2
Examination item	Number. of teeth	25 (7–32)	25 (7–32)	24 (7–31)	24 (7–31)	***	***	*
	Mean of PD (mm)	3.0 (2–4.3)	2.8 (2.2–4.1)	2.6 (2.1–3.4)	2.5 (2–3.6)	***	***	***
	Rate of PD 4–5 mm (%)	8.15 (0–62.9)	4.25 (0–42.1)	2.30 (28.1)	1.20 (0–35.7)	***	***	*
	Rate of PD ≥ 6 mm (%)	2 (0–27.3)	0.95 (0–26.5)	0 (0–15.5)	0 (0–11.9)	***	***	N.S.
	BOP rate (%)	15.3 (0–67.9)	12.4 (0–20.5)	7.7 (0.7–44.0)	10.4 (0–41.7)	***	N.S.	*
	PISA (mm^2)	234.1 (0–1929.2)	147.9 (0–1537.1)	113.8 (5.9–585.8)	126.3 (0–648.4)	***	**	*
	PESA (mm^2)	1410.0 (576.9–2768.9)	1246.9 (392.2–2653.2)	1150.8 (283.1–1791.8)	1044.8 (293.0–1742.4)	***	***	***
	PISA/PESA	0.20 (0–0.73)	0.15 (0–0.64)	0.10 (0.01–0.53)	0.12 (0–0.43)	***	*	***
	PCR (%)	35.7 (8.7–421.6)	30.0 (7.4–78.9)	33.3 (6.5–75.0)	34.0 (2.9–96.9)	*	*	N.S.
	Number of missing molars	3.0 (0–12.0)	3.0 (0–13.0)	3.5 (0–15.0)	4.0 (0–15.0)	***	***	***
	Eichner classification	3 (1–9)	4 (1–9)	4 (1–9)	4 (1–9)	***	***	***

A total of 106 patients were included in this study. The profiles of the periodontal parameters of the patients were demonstrated at four stages of periodontal therapy. The numbers in each box indicate the median (minimum – maximum). Wilcoxon signed-rank tests were used for the evaluations between BL, RE, SPT1, and SPT2. Statistically significant differences. (* $p < 0.05$, ** $p < 0.01$, *** $p < 0.001$). Abbreviations: PD, probing depth; BOP, bleeding on probing, PISA, periodontal inflamed surface area; PESA, periodontal epithelial surface area; PCR, plaque control record; BL, baseline; RE, reevaluation; SPT, supportive periodontal therapy.

Table S2. Details of the lifestyle habits questionnaire

	Median	Minimum	Maximum
Environmental factors			
Age	73	44	88
Smoking	0	0	2
Brinkman (Br) index	0	0	1500
BMI (kg/m^2)	23.0	15	37.6
Nutritional factors			
Rice	7	1	7
Bread	5	1	7
Noodles	3	1	6
Chicken	4	1	7
Pork, Beef, Mutton	4	1	7
Processed meat	3	1	7
Liver	1	1	6
Squid, Octopus, Shrimp, Shellfish	2	1	6
Fatty fish	3	1	7
Nonfatty fish	3	1	6
Egg	5	1	7
Soy	5	1	7
Tofu	4	1	7
Natto	4	1	7
Milk	5	1	7
Yogurt	6	1	7
Dark green vegetables	5	1	7
Cabbage, Napa cabbage	5	1	7
Carrot, Squash	4	1	7
Radish, Turnip	4	1	7
Other vegetables	5	1	7
Seaweed	4	1	7
Mushrooms	4	1	7

Smoking, one of the explanatory variables, was converted into three scores. Nutritional factors in the questionnaire were divided into seven scores based on frequency.

Table S3. Lifestyle habits questionnaire including environmental and nutritional factors

Environmental factors	Frequency	2		1		0		
Smoking	Explanation	Current smoker		Former smoker		Non smoker		
Br index	Number of cigarettes (/day) × year							
Nutritional questionnaire	Frequency	7	6	5	4	3	2	1
Each item	Explanation	Not less than twice a day	Once a day	4–6 times a week	2 or 3 times a week	Once a week	Less than once a week	No

The numbers in each box indicate the median (minimum – maximum). Abbreviations: BMI, body mass index; Br index, Brinkman index.

Figure S1. Histograms of environmental factors according to the results of the questionnaire survey. The vertical axes show the numbers, and the horizontal axes show each unit. Abbreviations: BMI, body mass index; Br index, Brinkman index.

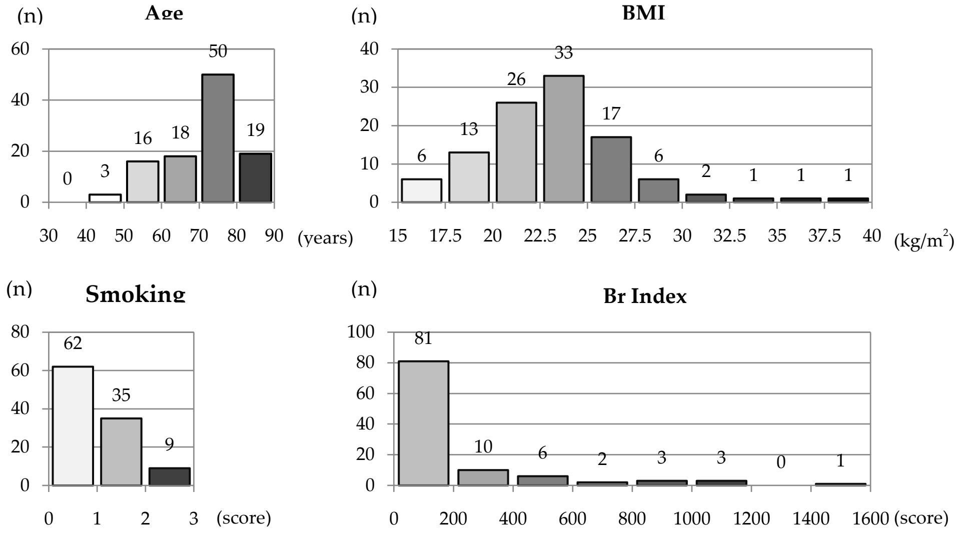

Figure S2. Histograms of nutritional factors according to the results of the questionnaire survey. The vertical axes show the numbers, and the horizontal axes show each unit

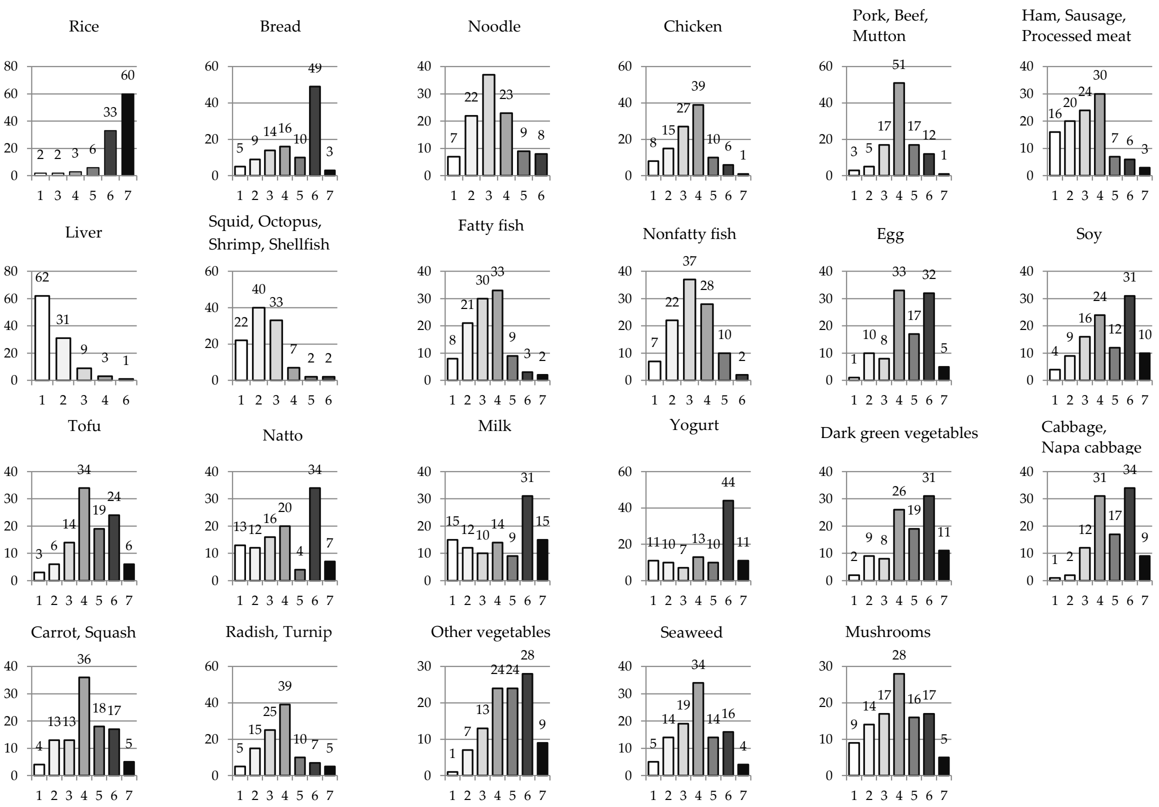

Figure S3. Residual plot based on the results of the Breusch–Pagan test and White's test for heteroskedasticity in the multiple regression analysis using environmental factors as the explanatory variables. The vertical axes indicate the residual of each environmental factor, and the horizontal axes indicate sample numbers in all graphs.

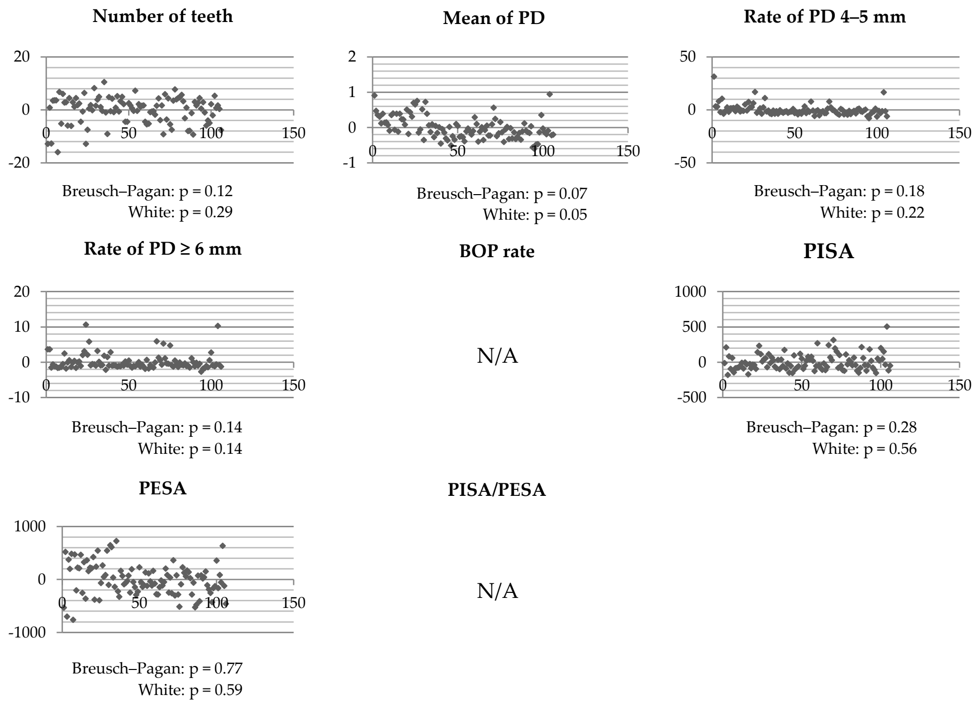

Figure S4. Residual plot based on the results of the Breusch–Pagan test and White's test for heteroskedasticity in the multiple regression analysis using nutritional factors as the explanatory variables The vertical axes indicate the residual of each nutritional factor, and the horizontal axes indicate sample numbers in all graphs.

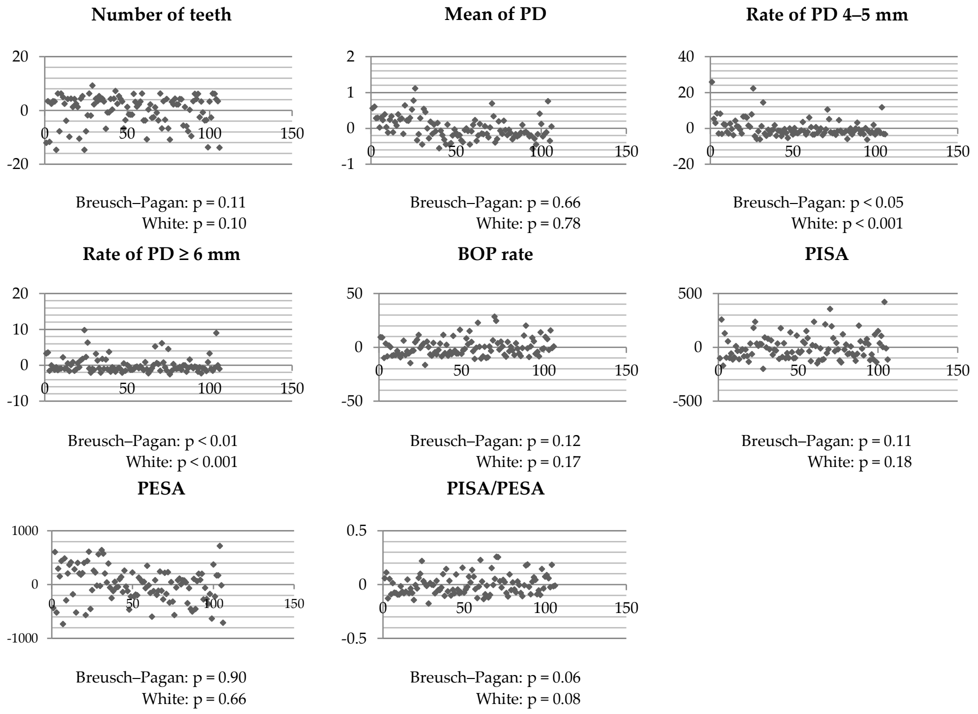

Supplement: Supplementary file 1 [file nutrients-14-04993-s001.zip › nutrients-2034710-SM .pdf]
